# Enhancing early-season soybean identification through optical and SAR time-series integration

**DOI:** 10.3389/fpls.2025.1656628

**Published:** 2025-10-20

**Authors:** Hongchi Zhang, Dailiang Peng, Changyong Dou, Zihang Lou, Xiaoyang Zhang, Le Yu, Kaishan Song, Yaqiong Zhang, Jinkang Hu, Shijun Zheng, Yulong Lv, Shengyi Liu, Yizhou Zhang, Hao Peng

**Affiliations:** ^1^ Key Laboratory of Digital Earth Science, Aerospace Information Research Institute, Chinese Academy of Sciences, Beijing, China; ^2^ International Research Center of Big Data for Sustainable Development Goals, Beijing, China; ^3^ College of Resource and Environment, University of Chinese Academy of Sciences, Beijing, China; ^4^ Institute of Agricultural Remote Sensing and Information Technology Application, College of Environmental and Resource Sciences, Zhejiang University, Hangzhou, China; ^5^ Geospatial Sciences Center of Excellence, Department of Geography Geospatial Sciences, South Dakota State University, Brookings, SD, United States; ^6^ Department of Earth System Science, Tsinghua University, Beijing, China; ^7^ State Key Laboratory of Black Soils Conservation and Utilization, Northeast Institute of Geography and Agroecology, Chinese Academy of Sciences, Changchun, China; ^8^ Satellite Application Center for Ecology and Environment, Ministry of Ecology and Environment, Beijing, China; ^9^ College of Forestry, Shenyang Agricultural University, Shenyang, Liaoning, China; ^10^ State Key Laboratory of Desert and Oasis Ecology, Xinjiang Institute of Ecology and Geography, Chinese Academy of Sciences, Urumqi, Xinjiang, China

**Keywords:** soybean mapping methods, remote sensing, Sentinel-1/2, time-series analysis, early-season crop identification

## Abstract

Soybean is an important grain and cash crop in China, and timely knowledge of its distribution is crucial for food security. However, traditional survey methods are time-consuming and limited in coverage. In contrast, satellite remote sensing enables large-scale, continuous, and cost-effective monitoring, providing reliable support for crop classification and yield forecasting. However, the high spectral similarity between soybean and maize during key phenological stages presents a major challenge for reliable classification. To address this, we propose a multi-source remote sensing approach that integrates Sentinel-1 SAR and Sentinel-2 optical time-series imagery. This method combines statistical descriptors, harmonic fitting parameters, phenological indicators, and radar-based features within a random forest classifier to achieve accurate soybean mapping. The study was conducted in the Jiusan Reclamation Area of Heilongjiang Province using satellite imagery from May to October 2019 for multi-source classification and temporal analysis. We systematically evaluated classification performance across different data sources and phenological stages and introduced the Earliest Identifiable Time (EIT) metric to assess temporal detection capabilities. Results show that the multi-source fusion approach outperforms single-source methods, achieving an overall accuracy (OA) of 96.85%, a Kappa coefficient of 0.9493, and an F1-score of 95.84% for soybean. Notably, SAR data significantly improved classification during the flowering stage—when optical imagery is often constrained—resulting in a maximum F1-score increase of 6.96%. Soybean classification accuracy increased rapidly with crop development, and the EIT was advanced to Day of Year (DOY) 210, approximately 20 days earlier than with optical data alone. These findings demonstrate the effectiveness of multi-source remote sensing in enhancing both the accuracy and timeliness of crop classification under complex climatic conditions, offering valuable support for precise soybean mapping and in-season monitoring.

## 1 Introduction

Soybean (Glycine max) is one of the most important plant-based protein crops worldwide. Its high protein and oil content have made it a globally cultivated crop, playing a central role in both food and feed systems (Schmutz et al., 2010; Liu et al., 2017). In China, soybean serves as both a staple and an economic crop, yet domestic production meets only a small fraction of national demand (She et al., 2024). Accurate and timely information on the spatial distribution of soybean cultivation is essential for assessing planting scale, informing policy implementation, and supporting efforts to boost domestic production (Johnson and Mueller, 2021; Wei et al., 2023).

Remote sensing has emerged as a powerful tool for large-scale, dynamic, and continuous land surface monitoring. It enables efficient identification and spatiotemporal analysis of agricultural features (Weiss et al., 2020; Bian et al., 2023), meeting the precision requirements of modern smart agriculture while reducing labor and financial input. By providing timely and consistent crop information from space, satellite technology helps stabilize supply chains, supports better policy decisions, and ultimately strengthens national and global food security. However, in major soybean-producing regions of China, soybean, maize, and rice are the dominant crops cultivated within the same agricultural landscape. Among these, soybean and maize exhibit high phenological and spectral similarity, especially during the early pod-setting stage, making their discrimination via single-source optical data extremely challenging (Chen et al., 2023; You et al., 2023; Zhang et al., 2024). Meanwhile, as a typical paddy crop, rice differs from upland soybean and maize in water demand and growth environment, yet its spectra can overlap with soybean in specific phenological phases, like seedling stage with sparse canopy, under cloud-contaminated optical data. Thus, developing high-accuracy and efficient mapping methods that simultaneously distinguish soybean from maize and rice is critical for optimizing cropping structures, increasing yields, and ensuring national food security (Li et al., 2023).

The increasing availability of free satellite imagery from platforms such as Sentinel-1 and Sentinel-2 has transformed agricultural monitoring, enabling high-resolution observations in spatial, temporal, radiometric, and spectral dimensions (Roy et al., 2014; Defourny et al., 2019; Khan et al., 2024). The integration of these data with advanced algorithms and cloud-based platforms like Google Earth Engine has made large-scale, field-level crop mapping more accessible and operational (Dong et al., 2016; Gorelick et al., 2017). Time-series remote sensing is particularly valuable for capturing dynamic crop phenology and reducing spectral confusion between similar crop types, such as maize and soybean, by leveraging the structured temporal signals embedded in satellite observations (You et al., 2021; Huang et al., 2024). Machine learning algorithms, especially Random Forest (RF), are widely used in crop classification due to their robustness, computational efficiency, and ability to handle high-dimensional, noisy data (Li et al., 2021; Song et al., 2021). RF performs well with time-series data and supports variable importance ranking, making it suitable for large-scale agricultural applications (You et al., 2021).

Although high-resolution optical imagery is effective for crop classification, especially at the end of the growing season, its utility is often limited in humid or rainy regions due to persistent cloud cover (Inglada et al., 2016; Jiao et al., 2022; Zhu et al., 2022; Maleki et al., 2024). Synthetic Aperture Radar (SAR), particularly from Sentinel-1, offers an all-weather, day-and-night imaging capability that ensures consistent data acquisition regardless of atmospheric conditions (Asam et al., 2022). SAR is sensitive to vegetation structure, biomass, and moisture content, complementing the spectral reflectance captured by optical sensors (Sun et al., 2019; Liao et al., 2020). For example, SAR can distinguish rice (with flooded fields in the early stage) from upland soybean/maize via specular reflection signals from water surfaces, while optical data excels at capturing chlorophyll-related spectral differences between soybean and maize. Numerous studies have demonstrated that combining Sentinel-1 and Sentinel-2 data significantly improves crop classification accuracy, often outperforming single-source methods (Gao et al., 2018; Moumni and Lahrouni, 2021; Li et al., 2022). This fusion leverages the strengths of both sensors: optical data effectively reflect vegetation activity and phenology, while SAR provides critical structural information and maintains coverage under cloud-obscured conditions (Qu et al., 2020; Huang et al., 2022; Xuan et al., 2023), leading to more robust characterization of crop dynamics.

Early-season crop type identification, mapping crops in early growth stages or before harvest, is crucial for supporting in-season decision-making (Inglada et al., 2016; You and Dong, 2020; Shen et al., 2025). Early classification benefits applications such as yield prediction, irrigation scheduling, and agricultural insurance, as well as land leasing and commodity trading (Konduri et al., 2020; Gallo et al., 2023; Rezaei et al., 2023). SAR’s all-weather observation capability is particularly valuable in the early season when optical imagery is frequently unavailable due to cloud cover (Wei et al., 2023; Zhou et al., 2024). Previous studies have shown that fusing SAR with optical data can achieve classification accuracy equivalent to that of optical-only data obtained a month later (Inglada et al., 2016), representing a significant advancement in early-season crop mapping.

Motivated by the increasing demand for accurate and timely information on soybean and recognizing the synergistic potential of multi-source time-series remote sensing, this study aims to enhance robust crop classification and early identification. Specifically, the objectives are: (1) to develop and evaluate an integrated multi-source time-series approach using Sentinel-1 SAR and Sentinel-2 optical data for accurate classification of major crops (rice, maize, and soybean), with a focus on improving soybean mapping; and (2) to investigate the temporal evolution of classification accuracy across the growing season, with the goal of determining the earliest identifiable time—defined as the point at which the soybean F1-score exceeds 0.9 (You and Dong, 2020). Additionally, we aim to quantify the incremental contribution of SAR data in improving classification accuracy and advancing the earliest identification window, thereby highlighting its value in supporting mid-season agricultural monitoring and decision-making.

## 2 Materials and methods

### 2.1 Study area

**Figure 1:**
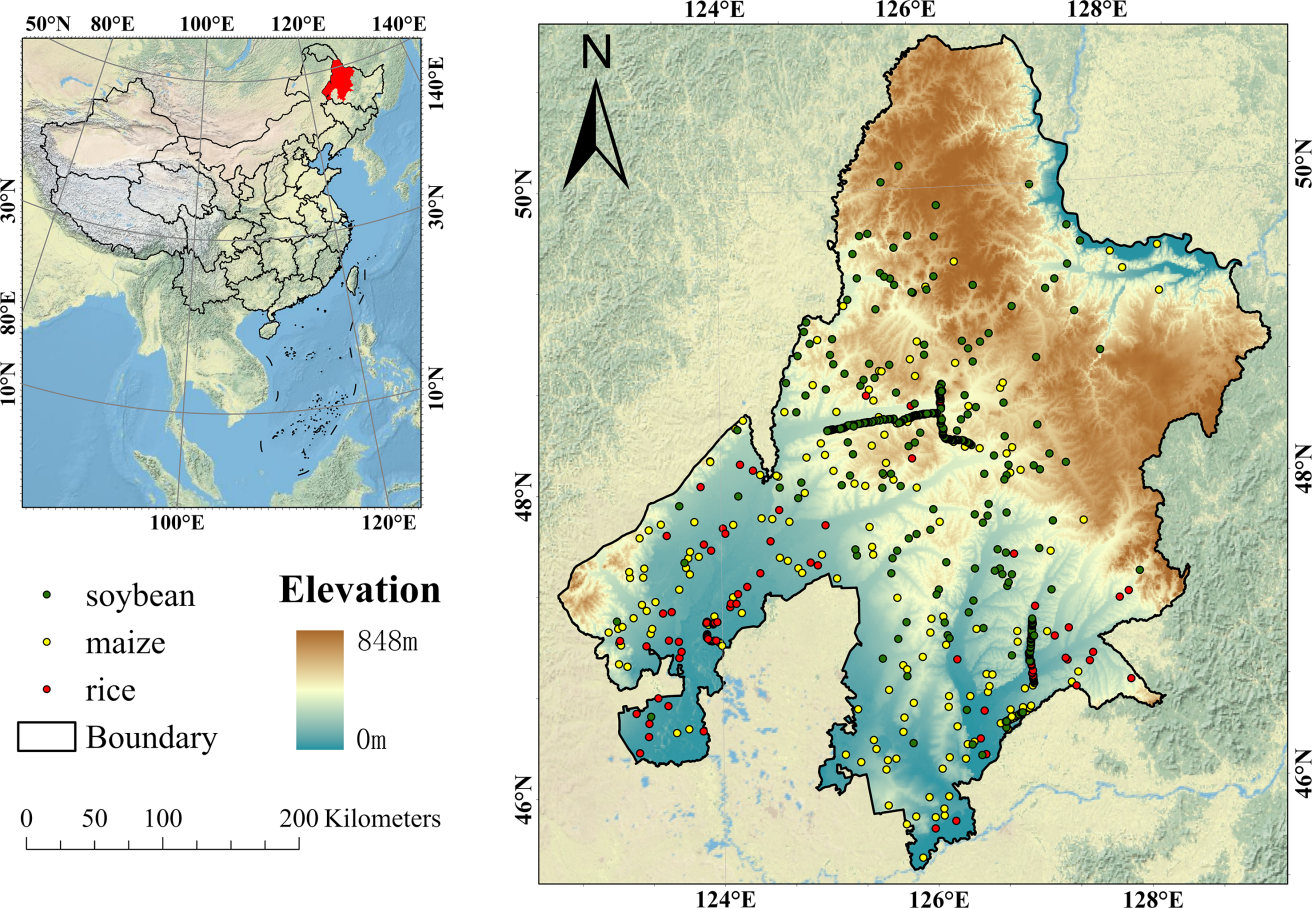
Geographical location of Jiusan Reclamation Area.

The Jiusan Reclamation Area is situated in the northwestern part of Heilongjiang Province, China, covering parts of Heihe, Suihua, and Qiqihar. Geographically, it spans from 122°24′E to 129°31′E longitude and from 45°30′N to 51°00′N latitude. The region features gently undulating terrain, forming a transitional zone between the Lesser Khingan Mountains and the Songnen Plain. Elevation ranges from 0 to 848 meters, with a general slope from northeast to southwest. The area experiences a mid-temperate, semi-humid continental monsoon climate, characterized by dry, windy springs and warm, humid summers. Annual precipitation ranges from 400 to 550mm, primarily concentrated between June and September. This climate regime—marked by the coincidence of heat and rainfall and large diurnal temperature variation—is highly conducive to crop growth and dry matter accumulation. The Jiusan Reclamation Area is a key agricultural production base in China, particularly for soybean, maize, and rice. Soybean has been cultivated in the region for decades with consistently stable yields, while maize acreage has expanded in recent years. Rice cultivation is concentrated in peripheral areas with sufficient water availability. According to agricultural statistics from Heilongjiang Province, Jiusan ranks among the leading regions nationwide in terms of soybean planting area and total production, playing a vital role in ensuring national food security. The geographic location of the Jiusan Reclamation Area is shown in **Figure 1**.

### 2.2 Datasets

#### 2.2.1 Sentinel-2 imagery and preprocessing

The Sentinel-2A and Sentinel-2B satellites, operated by the European Space Agency (ESA), are equipped with the MultiSpectral Instrument (MSI), which captures imagery at spatial resolutions ranging from 10 to 60 meters and provides a revisit frequency of five days. The MSI records data across 13 spectral bands, covering the visible to shortwave infrared (SWIR) regions (Immitzer et al., 2016). In this study, we uniformly resampled all Sentinel-2 bands to 10 meters spatial resolution for consistency in multi-source data fusion and crop classification—specifically, using 10-meter resolution bands (Blue, Green, Red, NIR) directly, and resampling 20-meter bands (Red Edge 2, SWIR1, SWIR2) to 10 meters via bilinear interpolation. We employed Level-2A surface reflectance (SR) products to identify soybean cultivation in the Jiusan Reclamation Area. These products have been publicly accessible via the Google Earth Engine (GEE) data catalog since 2019. To mitigate cloud contamination, the QA60 band—a bitmask containing cloud and cirrus detection information—was used to mask out opaque clouds and cirrus. All available Sentinel-2 images acquired between May and October 2019 with cloud cover below 50% were considered. Multiple satellite orbits were included to ensure complete spatial coverage of the study area. The number of observations varied across locations, with overlapping orbital paths resulting in denser temporal coverage in certain regions. In some cases, multiple acquisitions were made on the same day at different times, potentially introducing spectral variability. Furthermore, uneven image availability due to cloud cover and masking criteria led to irregular temporal intervals between usable observations. To generate a consistent time series for each pixel, 10-day median composites were created using all valid observations within each period (Amani et al., 2020). For regions lacking high-quality data due to persistent cloudiness or other issues, linear interpolation was applied using adjacent time steps to fill temporal gaps.

#### 2.2.2 Sentinel-1 imagery and preprocessing

The Sentinel-1A and Sentinel-1B satellites are equipped with C-band dual-polarization synthetic aperture radar (SAR) sensors, capable of acquiring imagery with spatial resolutions ranging from 5 to 40 meters and a revisit interval of 12 days. Sentinel-1 supports four imaging modes: Stripmap (SM), Interferometric Wide Swath (IW), Extra Wide Swath (EW), and Wave Mode (WV). Among these, the IW mode is commonly used for land applications and provides dual-polarization data, specifically vertical transmit–vertical receive (VV) and vertical transmit–horizontal receive (VH) polarizations. In this study, we used Sentinel-1 Ground Range Detected (GRD) products acquired in IW mode, which have a spatial resolution of 10 meters (azimuth) × 20 meters (range); these were further resampled to 10 meters spatial resolution (consistent with Sentinel-2) using nearest-neighbor interpolation to enable pixel-level multi-source data fusion. The preprocessing workflow included the following steps: (1) thermal noise removal; (2) radiometric calibration; (3) terrain correction using Shuttle Radar Topography Mission (SRTM) or ASTER digital elevation models (DEMs); and (4) conversion of terrain-corrected backscatter coefficients into decibel (dB) values. To further suppress speckle noise inherent in SAR imagery, a Refined Lee filter with a 7×7 moving window was applied (Yommy et al., 2015). To ensure temporal alignment with Sentinel-2 optical data, 10-day composite images were generated by calculating the median value of all valid Sentinel-1 observations within each corresponding period.

### 2.3 Auxiliary data

#### 2.3.1 Crop samples

This study collected a total of 1,424 ground sample points for soybean, maize, and rice in the study area for the year 2019, including 621 soybean samples, 588 maize samples, and 215 rice samples. These samples were collected with a mobile GIS device and subsequently checked against high-resolution Google Earth imagery and two seasonal Sentinel-2 RGB composites. Samples with mislabeling or located on roads and field boundaries were removed. The dataset was randomly divided into training and validation sets with a ratio of 8:2. A cropland mask was applied to exclude non-cropland pixels and prevent their inclusion in the crop classification process. Both the crop sample data and the cropland mask used in this study were derived from the 2019 Cropland Data Layer (CDL2019) for Northeast China (You et al., 2021).

#### 2.3.2 Statistical yearbook data

To validate the estimated soybean planting areas, official statistical data for 2019 soybean cultivation areas in Heihe, Suihua, and Qiqihar were obtained from the 2020 Heilongjiang Statistical Yearbook. For area accuracy assessment, the classified 10m resolution soybean maps were aggregated within Google Earth Engine using the pixelArea function to derive the total soybean area. The absolute difference between the remote sensing–derived soybean area and the official statistical yearbook records was then calculated to quantify area estimation error.

### 2.4 Methodology

**Figure 2:**
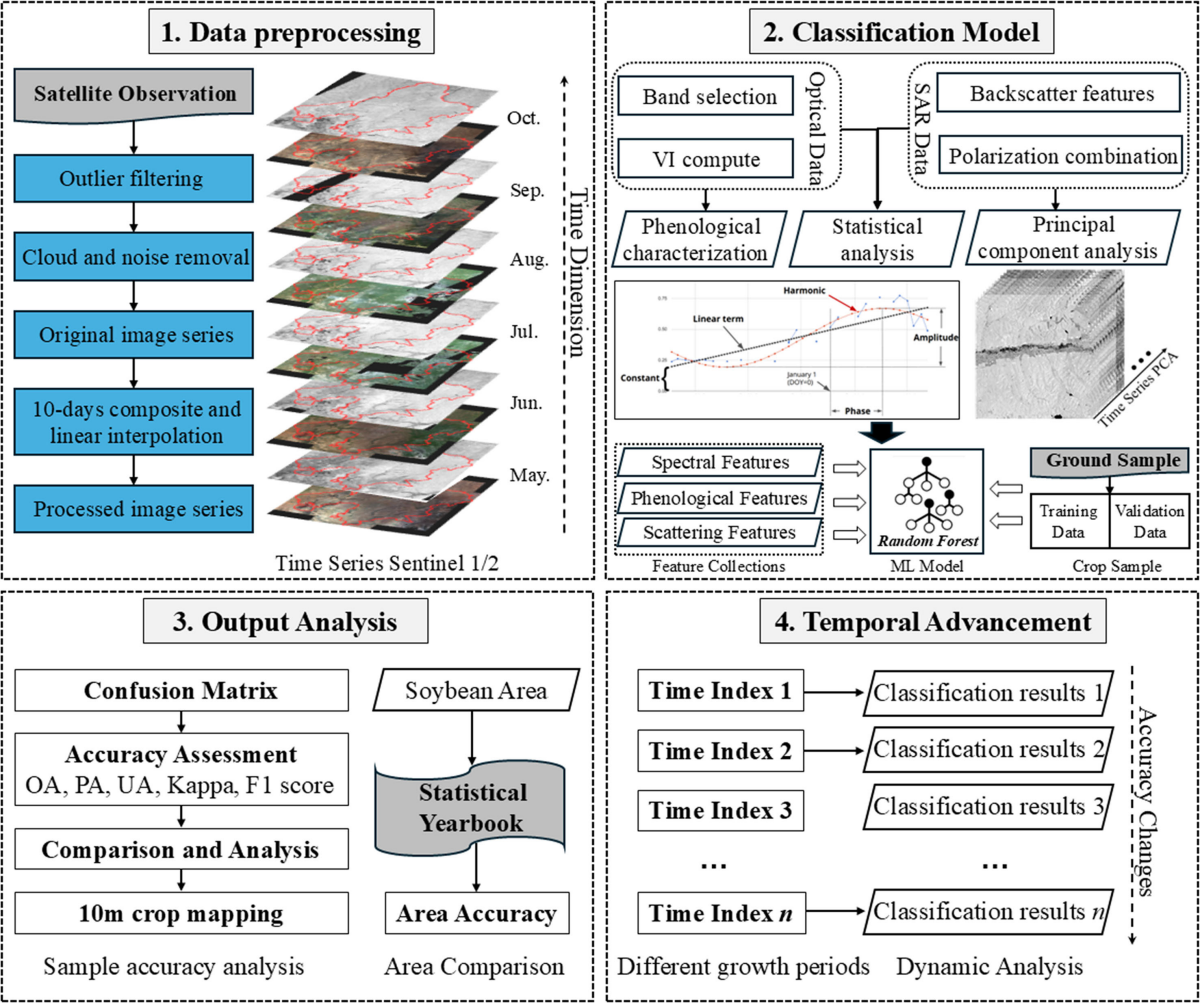
Research framework.

The research framework of this study is illustrated in **Figure 2** and consists of four main components: (1) Data Preprocessing: Sentinel-1 and Sentinel-2 time-series imagery was acquired and processed to remove outliers and cloud contamination, resulting in a clean and temporally consistent dataset. Smooth, continuous time series were constructed through 10-day median compositing and linear interpolation. (2) Classification Model Development: Features were extracted from both optical and SAR data, including spectral bands, vegetation indices, backscatter coefficients, and polarization metrics. These were integrated with phenological indicators derived from the time series of EVI calculated from Sentinel-2 imagery, which capture the timing of crop growth onset, senescence, and season length. In addition, statistical descriptors and principal component analysis (PCA) outputs of the Sentinel-1 backscatter time series (VV, VH, and related indices) were included to reduce redundancy while retaining key temporal variations. Together, these features formed a comprehensive dataset for classification. A Random Forest classifier was then trained using ground-truth samples for crop type identification. (3) Result Analysis and Accuracy Assessment: Classification performance was evaluated using confusion matrices and five standard metrics. Overall accuracy (OA) reflects the proportion of correctly classified samples. Producer’s accuracy (PA) measures omission errors, while user’s accuracy (UA) measures commission errors. The Kappa coefficient adjusts OA by accounting for chance agreement. The F1-score, as the harmonic mean of PA and UA, provides a balanced measure particularly useful when class sizes are uneven. Together, these metrics give a comprehensive evaluation of classification performance. Soybean planting area was estimated based on a 10-meter resolution classification map and compared with official statistical yearbook data to validate area estimation accuracy. (4) Time-Series Dynamic Analysis: To assess how classification accuracy evolved with crop growth, we designed a temporal progression experiment. Starting from May 1 (early sowing period), image sequences were gradually extended in 10-day steps until October 8 (harvest), resulting in 14 datasets. For each sequence, classification was performed and evaluated using the same independent validation set. This procedure allowed us to track temporal changes in accuracy and to determine the Earliest Identifiable Time (EIT), defined as the first date when the soybean F1-score exceeded 0.9.

#### 2.4.1 Parameter selection

To improve crop classification performance, this study selected a set of parameters for feature extraction based on the physiological and biochemical characteristics of soybean growth. Two categories of spectral data were employed to classify soybean and other crop types: (1) reflectance values from six spectral bands, and (2) values from five vegetation indices (**Table 1**). The six spectral bands included Red (492.4 nm), Green (559.8 nm), Blue (664.6 nm), Red Edge 2 (RE2, 740 nm), Shortwave Infrared 1 (SWIR1, 1610 nm), and Shortwave Infrared 2 (SWIR2, 2190 nm). Previous studies have highlighted the potential of SWIR1, SWIR2, and RE2 bands in effectively distinguishing between maize and soybean (Luo et al., 2021; Chen et al., 2023). In addition, six widely used spectral indices were calculated: Enhanced Vegetation Index (EVI), Land Surface Water Index (LSWI), Red Edge Position Index (REPI), Red Edge Normalized Difference Vegetation Index (RENDVI), and Normalized Difference Senescence Vegetation Index (NDSVI). The EVI time series is commonly used to extract temporal and phenological features for various crops. LSWI, which is sensitive to both leaf and soil moisture, is particularly effective for distinguishing rice from upland crops such as maize and soybean. REPI and RENDVI, which utilize Sentinel-2 red edge bands, are well-suited for estimating canopy chlorophyll content and nitrogen levels.

**Table 1:** Optical vegetation index.

Indices	Formulation*
EVI	$\text{EVI}=2.5\times \frac{{\text{ρ}}_{NIR}-{\text{ρ}}_{red}}{{\text{ρ}}_{NIR}+6\times {\text{ρ}}_{red}-7.5\times {\text{ρ}}_{blue}+1}$
LSWI	$\text{LSWI}=\frac{{\text{ρ}}_{NIR}-{\text{ρ}}_{SWIR1}}{{\text{ρ}}_{NIR}+{\text{ρ}}_{SWIR1}}$
REPI	$\text{REPI}=705+35\times \frac{\left({\text{ρ}}_{red}+{\text{ρ}}_{RE3}\right)/2-{\text{ρ}}_{RE1}}{{\text{ρ}}_{RE2}-{\text{ρ}}_{RE1}}$
RENDVI	$\text{RENDVI}=\frac{{\text{ρ}}_{NIR}-{\text{ρ}}_{RE2}}{{\text{ρ}}_{NIR}+{\text{ρ}}_{RE2}}$
NDSVI	NDPI=ρSWIR1−ρRedρSWIR1+ρRed

*ρblue, ρgreen, ρred, ρRE1, ρRE2, ρRE3, ρNIR, and ρSWIR1 represent the surface reflectance of Sentinel-2 MSI bands, corresponding to Band 2 (blue, 496.6 nm (S2A)/492.1 nm (S2B)), Band 3 (green, 560 nm (S2A)/559 nm (S2B)), Band 4 (red, 664.5 nm (S2A)/665 nm (S2B)), Band 5 (Red Edge 1, 703.9 nm (S2A)/703.8 nm (S2B)), Band 6 (Red Edge 2, 740.2 nm (S2A)/739.1 nm (S2B)), Band 7 (Red Edge 3, 782.5 nm (S2A)/779.7 nm (S2B)), Band 8A (NIR, 864.8 nm (S2A)/864 nm (S2B)), and Band 11 (SWIR1, 1613.7 nm (S2A)/1610.4 nm (S2B)), respectively.

To improve soybean identification accuracy under cloudy conditions, this study selected five key parameters from Sentinel-1 VV/VH dual-polarized SAR imagery to capture crop structural and physical characteristics across different growth stages (**Table 2**). These SAR features include the backscatter coefficients of the VV and VH polarization channels ( ${\text{σ}}_{\text{VV}}^{0}$ and ${\text{σ}}_{\text{VH}}^{0}$ ), their combined forms, and the dual-polarization Radar Vegetation Index (RVI) derived from these channels. The ${\text{σ}}_{\text{VV}}^{0}$ and ${\text{σ}}_{\text{VH}}^{0}$ coefficients reflect the radar backscattering response from vertical and horizontal plant structures, respectively (Pageot et al., 2020). VV polarization is more sensitive to vertical canopy characteristics and is thus closely related to soybean biomass and canopy density, while VH polarization responds more strongly to variations in surface roughness and structural complexity, making it useful for detecting changes during mid to late phenological stages. RVI, a normalized index computed from the VV and VH channels, effectively characterizes crop canopy vigor and growth dynamics (Mandal et al., 2020). Additionally, the Cross-Polarization Ratio (CPR), defined as the ratio of the product to the sum of ${\text{σ}}_{\text{VV}}^{0}$ and ${\text{σ}}_{\text{VH}}^{0}$ , enhances sensitivity to backscatter intensity during key phenological events such as the pod-filling stage (Veloso et al., 2017). Collectively, these SAR-derived parameters provide essential physical and temporal insights that support robust soybean classification in multi-source remote sensing applications.

**Table 2:** SAR parameter index.

Parameters	Proxies	Description
Backscattering Ratio	${\text{σ}}_{\text{VH}}^{0},\;{\text{σ}}_{\text{VV}}^{0}$	Throughout the soybean growth period, alterations in the growth status and density of soybean leaves, stems, and pods can have substantial effects on the backscattering ratio (Pageot et al., 2020).
Cross-Polarization Ratio	$\frac{{\text{σ}}_{\text{VH}}^{0}*{\text{σ}}_{\text{VV}}^{0}}{{\text{σ}}_{\text{VV}}^{0}+{\text{σ}}_{\text{VH}}^{0}}$	Fluctuations over time in this index reflect changes in moisture content and structure that are associated with phenological development (Khabbazan et al., 2019).
RVI	$\text{RVI=}\frac{4\times {\text{σ}}_{\text{VH}}^{0}}{{\text{σ}}_{\text{VH}}^{0}+{\text{σ}}_{\text{VV}}^{0}}$	RVI can characterize both crop biomass and the LAI (Chang et al., 2018).

#### 2.4.2 Feature extraction

Based on the spectral curve dynamics of crops and incorporating phenology and spectral index information, this study extracted statistical features, peak growth period features, harmonic fitting features, phenological features, and SAR features to enhance the discrimination between soybeans and non-soybean crops, as summarized in **Table 3**.

**Table 3:** Summary of classification features.

Feature type	Feature name	Processing method	Quantity
Vegetation Indices Time Series	EVI, LSWI, RENDVI, REPI, NDSVI	min, max, std, and 15/50/90th percentile	5×6
Visible and Red-Edge Band Time Series	B2, B3, B4, B6 (490nm−740nm)		4×6
Shortwave Infrared Band Time Series	B11, B12 (1610nm−2200nm)		2×6
Phenological Features	SOS EOS LOS	Median method	3×1
EVI Time Series Features	(EVI) Phase and Amplitude	Harmonic fitting	1×2
Accumulated Biomass Features	EVI	Accumulation	1×1
Statistical Features	${\text{σ}}_{\text{VH}}^{0},\;{\text{σ}}_{\text{VV}}^{0},\text{ CPR},\text{ RVI}$	max, min, mean, stdv, 15/50/90th percentile	7×4
Principal Component Features	${\text{σ}}_{\text{VH}}^{0},\;{\text{σ}}_{\text{VV}}^{0},\text{CPR},\text{ RVI}$	Principal Component Analysis	3×4

1. Statistical Features and Peak Growth Period Features

Statistical features include the maximum, minimum, variance, and the 15th, 50th, and 90th percentiles of 11 spectral parameters throughout the entire growth season. The maximum and minimum values represent the upper and lower bounds of spectral variation, which differ significantly among crops and help distinguish soybeans from others. Variance reveals the fluctuation in spectral curves, reflecting the stability of the growth state. Percentiles provide spectral distribution information during early, middle, and late growth stages. All these statistics were calculated at the pixel level across the full time series, rather than aggregated by plot, which preserves intra-field variability and enables the detection of mixed conditions such as intercropping. Peak growth period features are extracted based on the timing of peak values in the EVI and LSWI indices. The corresponding “greenest” and “wettest” images are composited for these peak periods, from which the 11 spectral parameters are extracted as features representing the crop’s growth peak. This period typically coincides with soybean pod development, when its spectral behavior most notably differs from other crops, making these features especially critical for classification.

2. Harmonic Fitting Features

Harmonic fitting is an effective time series modeling method to extract periodic features. By treating the time series as a periodic function and applying Fourier transformation, the original curve can be reconstructed using several sine and cosine components. The function is expressed as:


$$f\left(t\right)=a_{0}+\sum_{k=1}^{n}\left[a_{k}\cos\left(2\pi k\omega t\right)+b_{k}\sin\left(2\pi k\omega t\right)\right]$$

where f(t) represents the fitted vegetation index value at time  t , $a_{0}$ is the constant term, $a_{k}$ and $b_{k}$ are the coefficients for cosine and sine terms respectively. *n* is the order of the harmonic fitting, and ω is the frequency, set to 1.5.t denotes the position of the current day of year (DOY) within the year, expressed as a decimal between 0 and 1.

3. Phenological Features

Phenological features directly reflect the timing of crop developmental stages from sowing to maturity and serve as important remote sensing parameters for representing crop growth processes. Different crops exhibit distinct spectral variation timings due to differences in planting dates and growth cycles, making phenological features effective classification criteria. The phenological features extracted include: (i) cumulative growing degree days for the start (SOS), end (EOS), and length (LOS) of the growing season; (ii) EVI values at SOS and EOS; (iii) cumulative EVI during the growing season as a proxy for accumulated biomass. These are extracted using a threshold method: the EVI time series is sorted and the median value is selected as the threshold. The first date when EVI exceeds this threshold marks SOS, the last date marks EOS, and LOS is the difference between the two. This approach suits single-cropping systems (e.g., soybean, maize, rice), where EVI typically shows a clear increase and decline pattern.

4. SAR Features

For the four SAR feature parameters, we leveraged the SAR image time series to extract key crop characteristics, including statistical and principal component features. The statistical features are consistent with those used for optical data, covering maximum, minimum, variance, and the 15th, 50th, and 90th percentiles of five SAR parameters. These statistics help convey the average level and temporal variation within crop-specific time series curves. Additionally, principal component analysis (PCA) was performed on the Sentinel-1 time series in the temporal domain, with the first three principal components selected as the SAR principal component features.

#### 2.4.3 Random forest

A Random Forest (RF) classifier was employed to identify soybean cultivation. As a non-parametric machine learning algorithm, RF offers strong fault tolerance and has been widely adopted in crop classification and mapping studies due to its robustness, accuracy, and efficiency. The algorithm is also well supported by the Google Earth Engine (GEE) platform, making it convenient for large-scale implementation. In this study, the RF model was configured using two key parameters: (1) numberOfTrees, which defines the number of decision trees in the ensemble. A larger value generally improves classification accuracy but increases computation time linearly; this parameter was set to 100. (2) minLeafPopulation, which specifies the minimum number of samples required at each leaf node. To reduce the risk of overfitting, it was set to 10. In addition to classification, the RF algorithm provides a measure of feature importance, enabling effective feature ranking and selection. Feature importance was assessed using the Mean Decrease in Impurity (MDI), which quantifies the reduction in node impurity contributed by each feature across all decision trees. Features contributing more to impurity reduction are considered more informative. Classification performance was evaluated using an independent test dataset. Standard accuracy metrics were reported, including overall accuracy (OA), producer’s accuracy (PA), user’s accuracy (UA), the Kappa coefficient, F1 score, and area-based accuracy.


$$OA=\frac{N_{S}+N_{O}}{n}$$

$$PA=\frac{N_{S}}{C_{S}}\;UA=\frac{N_{S}}{T_{S}}$$

$$F1\;score=\frac{UA\times PA}{UA+PA}\times 2$$

$$Area\;Accuracy=1-\frac{\left|Area_{gt}-Area_{rs}\right|}{Area_{gt}}$$

Where $N_{S}$ is the number of correctly classified soybean samples; $C_{S}\;$ is the total number of samples classified as soybean; $T_{S}$ is the total number of soybean validation samples; $N_{O}$ is the number of correctly classified non-soybean samples; n is the total number of all validation samples; $Area_{gt}$ represents the actual soybean planting area obtained from statistical yearbook data, while $Area_{rs}$ denotes the soybean planting area estimated through remote sensing classification.

## 3 Results and discussion

### 3.1 Spectral reflectance and backscattering characteristics of crops

**Figure 3:**
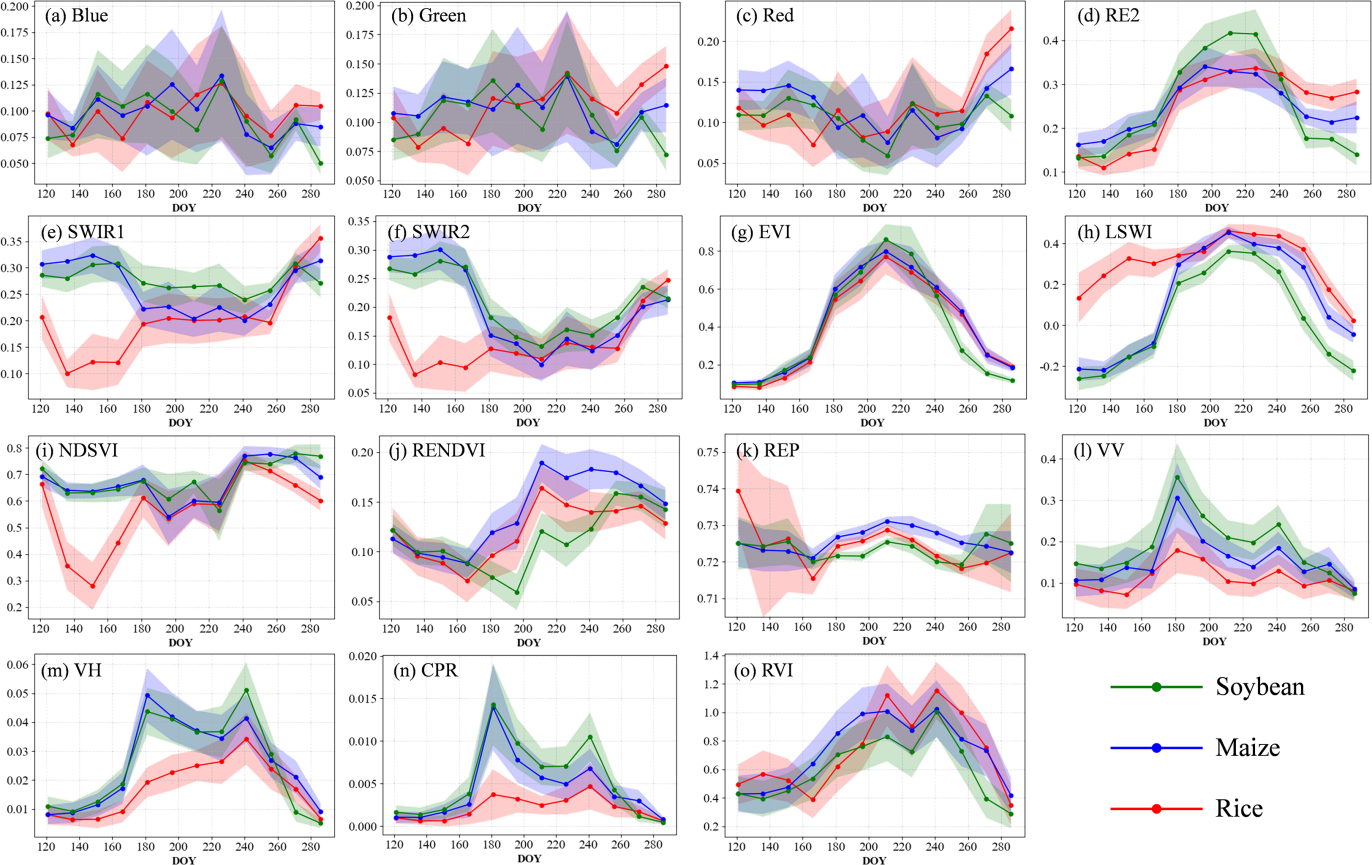
Time series curves of spectral reflectance **(a–f)**, vegetation indices **(g–k)**, and radar-based parameters **(l–o)** for soybean and other major crops. The curves are generated from all training samples within the study area. The x-axis represents the day of year (DOY), while the y-axis shows the values of spectral reflectance and vegetation indices. Solid lines indicate the mean values, and shaded areas represent one standard deviation.

**Figure 3** illustrates the time series curves of key spectral bands, vegetation indices, and SAR backscatter parameters used for crop classification, highlighting the seasonal dynamics of soybean, maize, and rice throughout the growing period. In the visible bands (Blue, Green, and Red), high standard deviations and substantial overlap among the three crops limit their discriminative power. Specifically, soybean and maize maintain relatively stable reflectance before DOY 220, followed by a rapid decline reaching a minimum around DOY 260. In contrast, rice exhibits a distinct pattern characterized by an initial decrease, a subsequent rise, and a sharp drop. Red band reflectance shows an inverted U-shaped trajectory, consistent across all crops, reflecting chlorophyll dynamics during the growth cycle. In the red-edge band (RE2), reflectance increases steadily during early growth and peaks around DOY 220. Soybean displays the highest RE2 values prior to this point but declines rapidly thereafter due to senescence, eventually falling below those of maize and rice. The SWIR1 and SWIR2 bands, which are sensitive to water content, show significantly lower reflectance for rice during the inundation phase (DOY 120–200), distinguishing it from upland crops. Between DOY 200 and 260, soybean reflectance in these bands remains consistently higher than maize, offering some potential for discrimination. For vegetation indices, both EVI and NDVI exhibit patterns similar to RE2, capturing the physiological transition from rapid vegetative growth to senescence. However, their time series also show substantial overlaps across crops. LSWI, which reflects canopy and surface water content, remains consistently high and stable for rice throughout the season, providing a reliable indicator for its identification. NDSVI shows a pronounced rise-then-fall trend in rice, with greater temporal variability than in soybean and maize, effectively capturing the phenological rhythm of rice development. REPI, which correlates with cumulative biomass, is higher in maize during the late season, indicating a red-edge shift associated with increased chlorophyll content. RENDVI values are notably higher for maize than for soybean and rice between DOY 200 and 260, offering improved separability, particularly between soybean and maize.

SAR-derived features (VV and VH backscatter) are influenced by both vegetation structure and moisture content and follow a general rise–fall pattern across all crops. Between DOY 120 and 140, rice exhibits a sharp decline in backscatter due to specular reflection from water surfaces, maintaining low values throughout the season, which distinctly separates it from upland crops. In the VV channel, soybean and maize backscatter gradually increase from DOY 120 to 200 as canopy moisture accumulates, then stabilize before declining sharply after DOY 260. In the VH channel, soybean shows consistently higher backscatter than maize between DOY 200 and 260, indicating a more complex canopy structure. The Radar Vegetation Index (RVI) peaks around DOY 200, with rice maintaining significantly lower values than soybean and maize, enabling strong crop discrimination. Soybean’s RVI also exhibits less temporal variability than maize, reflecting greater growth stability. The Cross-Polarization Ratio (CPR) further enhances classification accuracy; during the peak growth phase (DOY 220–240), soybean displays higher CPR values than both maize and rice, making it one of the most informative SAR-derived features for distinguishing soybean.

### 3.2 Soybean classification accuracy based on different data sources

**Figure 4:**
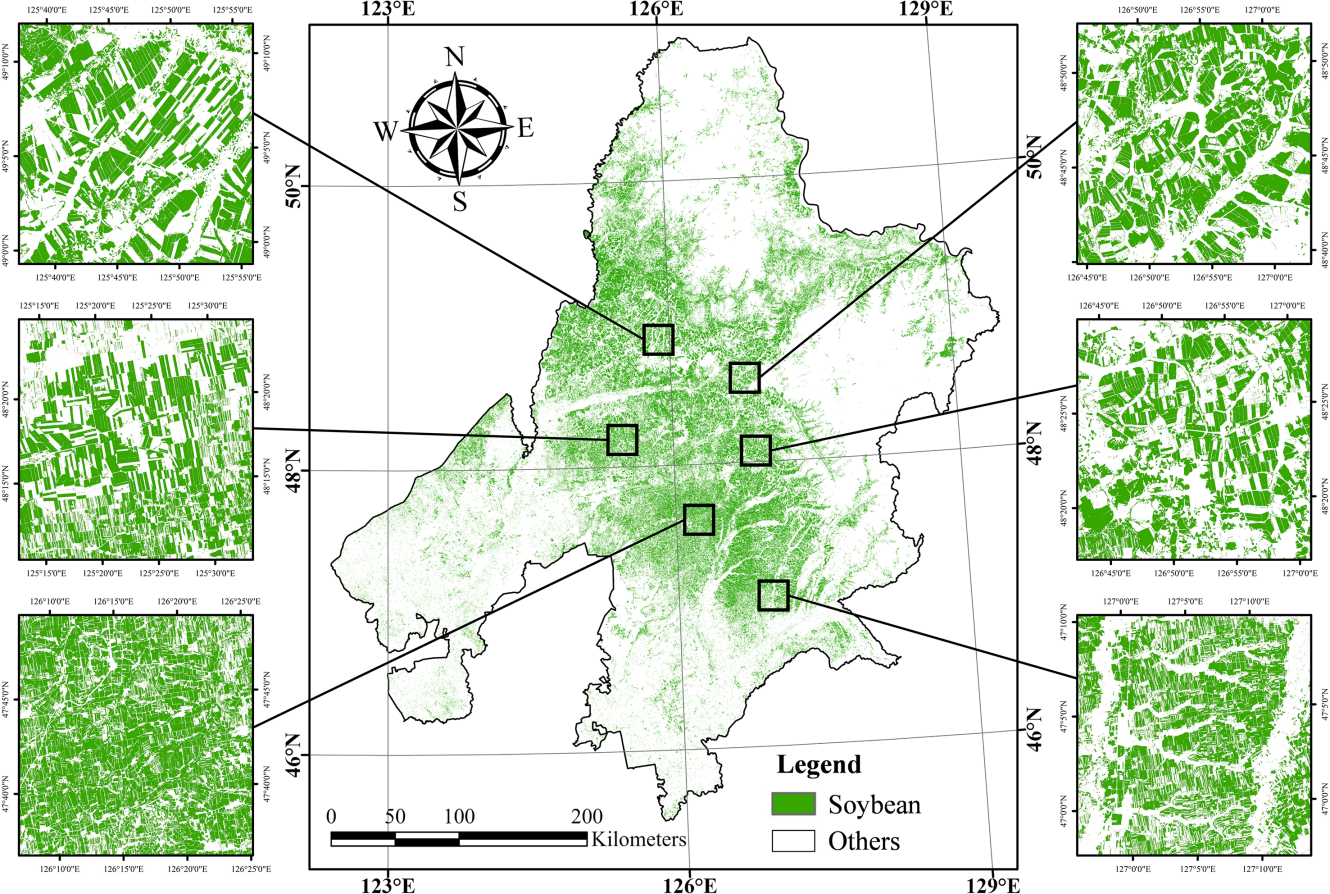
Identification and mapping of soybean planting areas in Jiusan Reclamation Area.

Using multi-source remote sensing data, crops in the study area were classified, and soybean planting areas were extracted. The classification achieved high accuracy, with an overall accuracy (OA) of 96.85%, a Kappa coefficient of 0.9493, and soybean-specific metrics of 98.30% for producer accuracy (PA), 93.51% for user accuracy (UA), 95.84% for F1-score, and 96.57% for area-based accuracy. The spatial distribution of soybean cultivation is illustrated in **Figure 4**.

Soybean planting was primarily concentrated between 46°N and 48°N, within a hilly transitional zone between the southern edge of the Lesser Khingan Mountains and the Songnen Plain—an area characterized by favorable agroecological conditions. The total soybean area in the study region reached 2.62 million hectares. Among the administrative divisions, Heihe recorded the largest planting area, with approximately 1.27 million hectares (48.33% of the total), mainly distributed across Nenjiang, Wudalianchi, and Beian, with smaller areas in the northern county of Sunwu. Qiqihar ranked second, with 0.83 million hectares (31.84%), primarily concentrated in eastern and northern counties including Nehe, Keshan, Kedong, Baiquan, and Yian, and with scattered fields in Gannan County in the west. Suihua accounted for 0.52 million hectares (19.83%), mainly located in the northern counties of Hailun, Suiling, and Wangkui. Spatially, soybean cultivation exhibited a relatively clustered distribution, though the field parcels were generally fragmented, small, and discontinuous. This pattern is influenced by natural topography, river networks, and the predominance of smallholder farming. In northern Qiqihar, soybean is often intercropped with maize, and fields tend to be narrow and elongated (approximately 60m × 1200m), with closely spaced rows. In contrast, northwestern Qiqihar and southwestern Heihe feature more regular and contiguous fields, typically rectangular and reaching up to 800m × 2400m. In the mountainous areas of central and northern Heihe, irregular field shapes are common due to the complex terrain. At the junction of Heihe, Qiqihar, and Suihua, a state-operated farming zone and the core area of intensive soybean cultivation. Fields in this region are generally square-shaped (approximately 400m × 400m), clearly delineated by internal road networks, with minimal intercropping. The planting system here is more large-scale and centralized, reflecting a more modern and industrialized agricultural structure.

**Figure 5:**
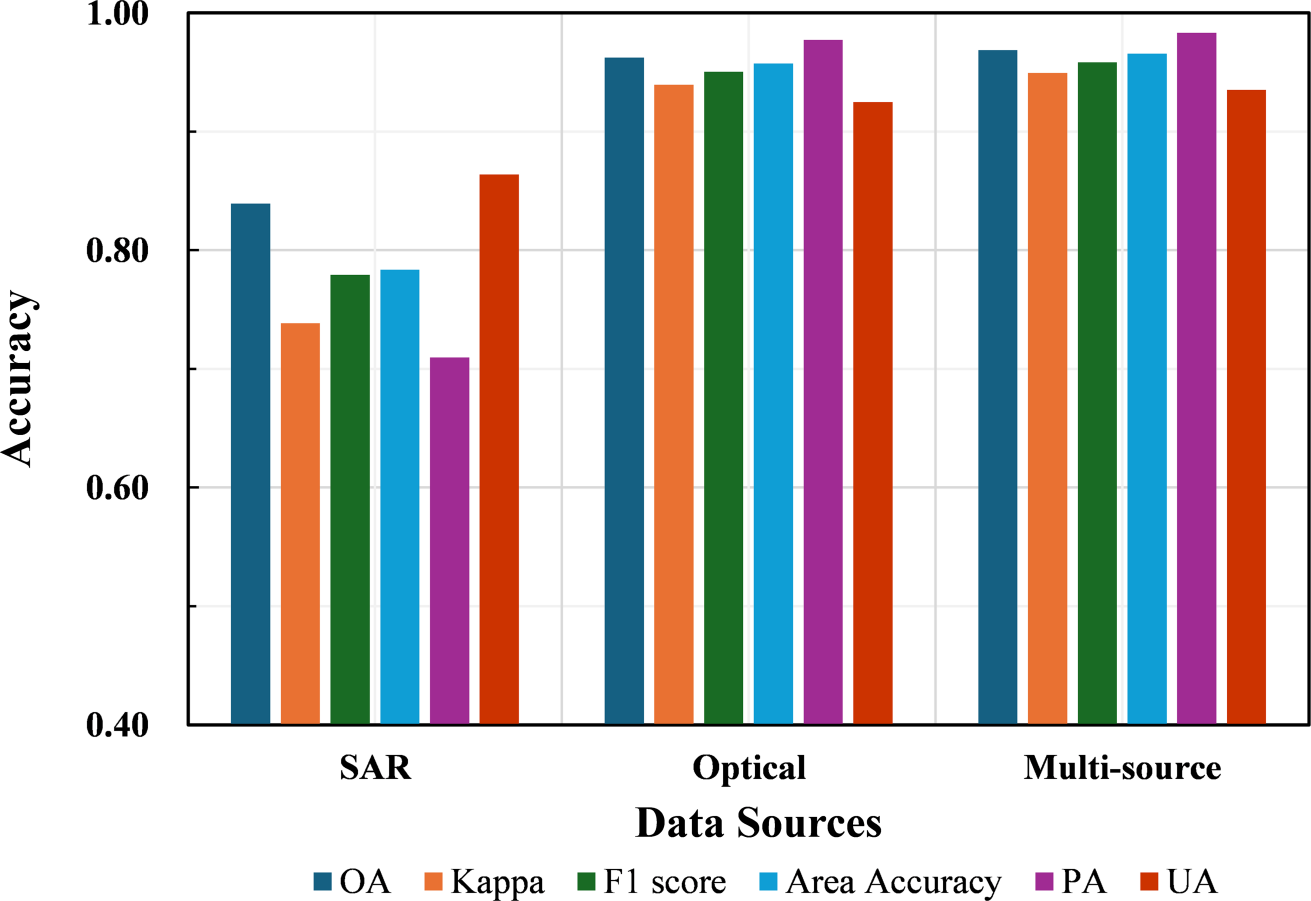
Accuracy results of different data sources.

In terms of classification performance using individual data sources (**Figure 5**), the overall accuracy (OA) and Kappa coefficient based solely on SAR data reached 83.92% and 0.7384, respectively. Although these values are lower than those achieved using optical data, SAR still exhibits a certain level of capability in crop discrimination. In contrast, the optical-only approach delivered significantly higher performance, with OA and Kappa values of 96.23% and 0.9394, respectively—second only to the multi-source fusion method—highlighting its strong ability to distinguish between crop types. When integrating both optical and SAR data, the multi-source fusion approach achieved the highest accuracy, with an OA of 96.85% and a Kappa coefficient of 0.9494. Compared with the optical-only approach, this represents an improvement of 0.62% in OA and 0.0099 in Kappa; compared with the SAR-only method, the improvement is more substantial—14.93% in OA and 0.2010 in Kappa. These results suggest that multi-source fusion can further enhance classification performance, even when starting from a high baseline, though the incremental gains over optical data alone are relatively modest.

From the perspective of soybean identification (**Figure 5**), the three data source strategies consistently followed the performance hierarchy: multi-source > optical > SAR. Evaluation metrics reveal that the SAR-only approach yielded notably higher user accuracy (UA) than producer accuracy (PA) for soybean, indicating a tendency toward underestimation, with the identified soybean area totaling only 1.985 million hectares. In contrast, both the optical and multi-source approaches exhibited higher PA than UA, suggesting more balanced classification outcomes. The F1 scores for soybean identification were 77.91% for SAR, 95.03% for optical, and 95.84% for the multi-source approach. Area-based accuracy followed a similar trend: 78.37% (SAR), 95.74% (optical), and 96.57% (multi-source). Compared to optical data, the multi-source fusion method achieved improvements of 0.81% in F1 score and 0.83% in area accuracy; when compared to SAR, these improvements rose to 17.93% and 18.20%, respectively. Overall, while multi-source data only slightly outperforms optical data, it substantially enhances performance over SAR, underscoring its value in robust and precise soybean identification.

**Figure 6:**
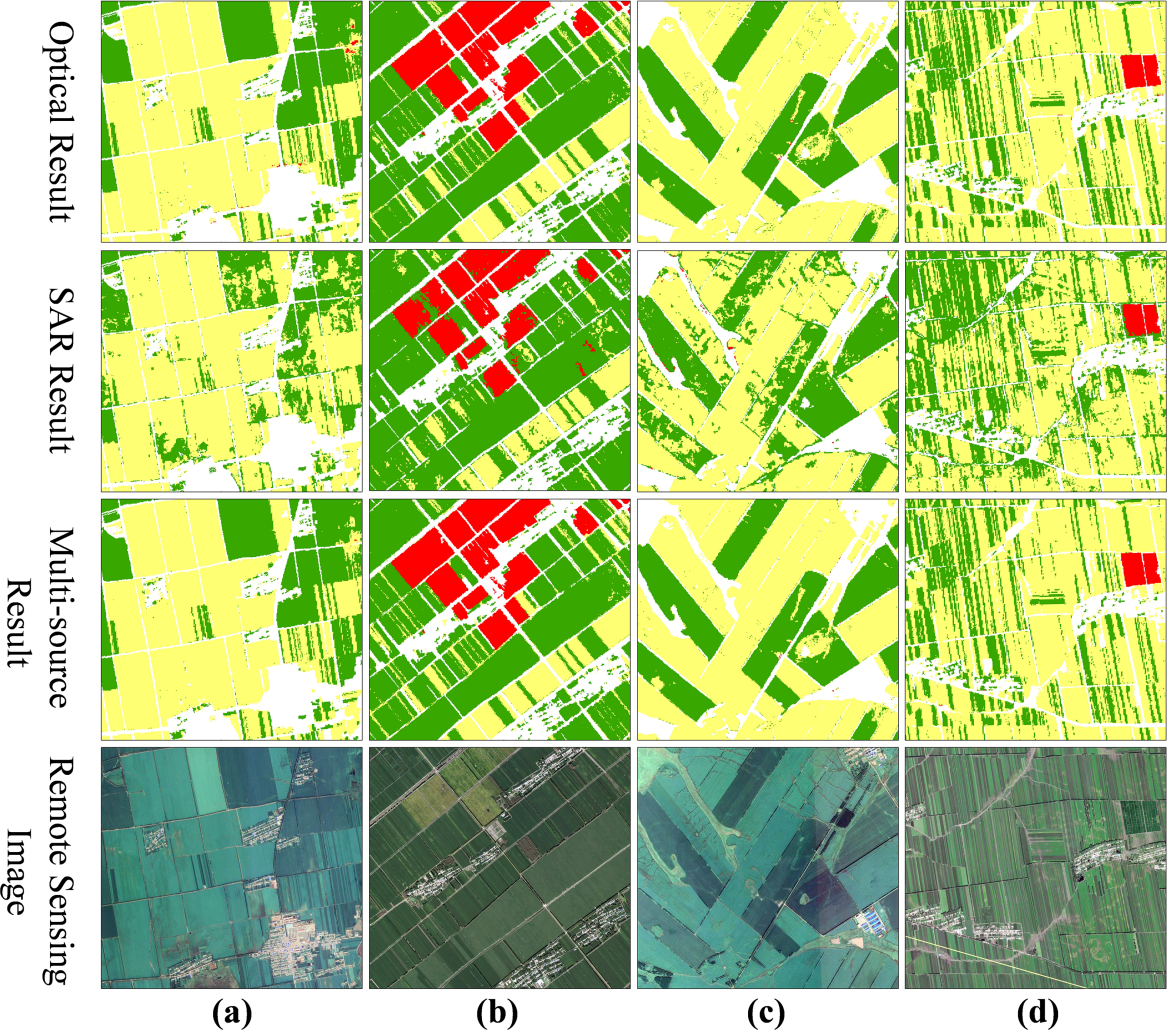
Comparison of local classification results under different data sources. **(a–d)** show four representative local scenes selected from different parts of the Jiusan Reclamation Area. For each scene, four maps are presented: the optical-only classification, the SAR-only classification, the multi-source classification, and the corresponding reference map derived from high-resolution Google Earth imagery. In the classification maps, soybean, maize, and rice are shown in green, yellow, and red, respectively.

**Figure 6** illustrates crop classification results for four selected local scenes, comparing outputs derived from optical data alone, SAR data alone, and a fusion of both data sources. Each classification map is accompanied by a corresponding reference map and high-resolution Google Earth imagery for validation. The optical-only classification already demonstrates strong performance, with clearly defined crop boundaries and complete field delineation. Even in regions where soybean and maize are intercropped, the model successfully achieves fine-scale differentiation between the two crops. In contrast, the SAR-only classification delivers more limited results, capturing only the general spatial patterns of crop distribution, but exhibiting considerable speckle noise and frequent misclassifications. The highest classification accuracy is attained using the multi-source fusion approach. Although the visual outcomes are broadly similar to those from the optical-only method, a detailed comparison reveals that the optical results still contain localized misclassification patches. These are significantly reduced in the multi-source results, leading to more precise delineation of crop field boundaries that align more closely with high-resolution reference imagery.

**Figure 7:**
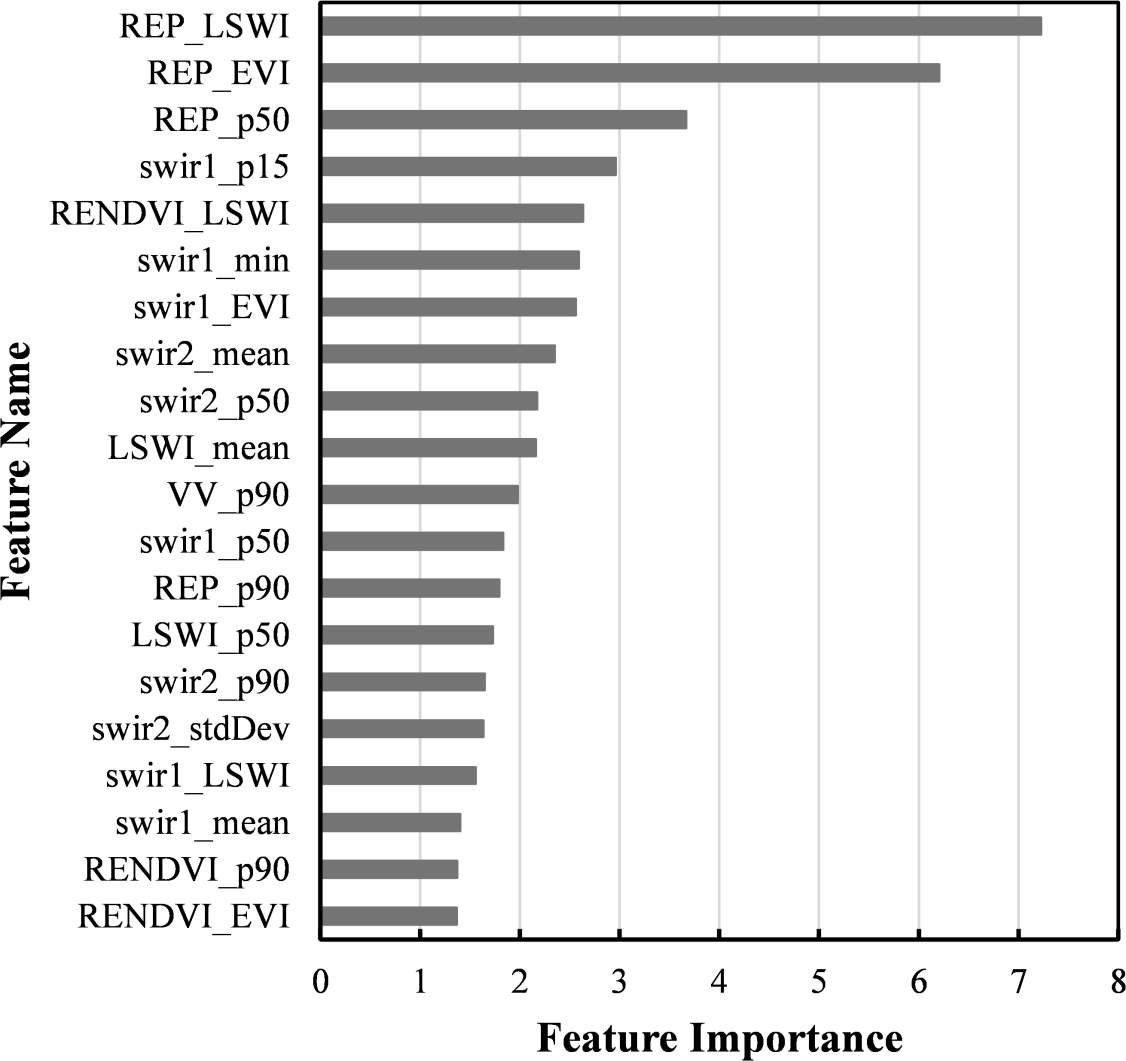
Ranking of features importance.

Feature selection was conducted using the Mean Decrease in Impurity (MDI) metric from the Random Forest classifier to assess the importance of each variable. Based on their importance scores, all features were ranked, and a subset comprising the top-ranked 20 features was identified as optimal (**Figure 7**). **Table 4** compares the classification performance of the full feature set and the selected subset. The results indicate that the subset yields classification accuracy largely comparable to that of the full set. The most notable difference is a roughly 1% decrease in user accuracy (UA) for soybean in the reduced feature set, resulting in a more pronounced overestimation of soybean area and a corresponding 3.4% decline in area-based accuracy. Minor reductions were also observed in overall accuracy (OA), Kappa coefficient, and soybean F1 score, each by approximately 0.3%. Notably, this reduction in feature dimensionality achieved an 87% decrease in data volume. Therefore, the selected 20-feature subset offers a favorable trade-off, maintaining high classification accuracy while significantly improving computational efficiency.

**Table 4:** Accuracy of full feature set and selected feature subset.

Feature set	OA	Kappa	Soybean PA	Soybean UA	Soybean F1 score	Soybean area accuracy
Full Feature Set	96.85%	0.9493	98.30%	93.51%	95.84%	96.57%
Selected Feature Set	96.63%	0.9460	98.86%	92.55%	95.60%	93.17%

The suffixes in the feature names denote specific types of information: “_EVI” and “_LSWI” represent feature values extracted during the respective peak periods of the EVI and LSWI indices; “_p15,” “_p50,” and “_p90” correspond to the 15th, 50th (median), and 90th percentiles; while “_min,” “_max,” “_mean,” and “_stdDev” denote common statistical measures, including minimum, maximum, mean, and standard deviation. The feature importance ranking indicates that REPI-related features occupy the highest positions, particularly those derived from the wettest and greenest composites, as well as median values. This highlights their critical role in crop classification, especially for soybean identification, and aligns well with the results from temporal curve analysis. Features from the shortwave infrared bands (SWIR1 and SWIR2) also demonstrate strong discriminative power, comprising 10 of the top 20 features. Additionally, LSWI and RENDVI features appear frequently, contributing to the effective identification of rice and to the differentiation between soybean and maize, respectively. Among SAR-derived features, only the 90th percentile of VV polarization is included in the optimal subset, suggesting the dominance of optical features within the multi-source dataset. This finding is consistent with previous studies reporting that Sentinel-1 and Sentinel-2 data contribute approximately 27% and 73%, respectively, to overall crop classification accuracy. Moreover, the frequent presence of growth peak features and 90th percentile values reflects that crops exhibit their greatest spectral separability during peak growth stages.

### 3.3 Changes in soybean recognition accuracy with crop growth and temporal progression

**Figure 8:**
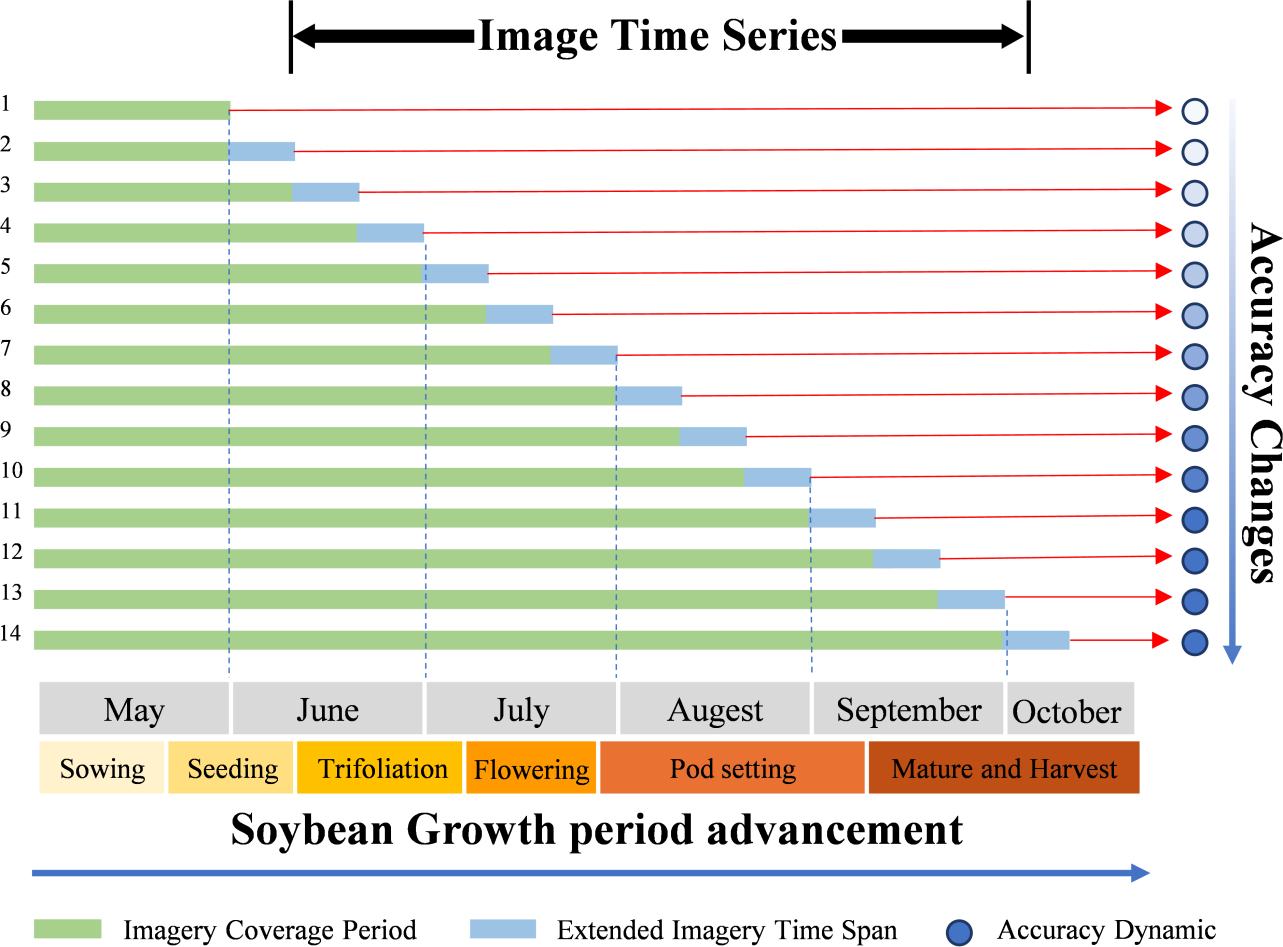
Experimental design for time-series progression analysis. Each row (1–14) represents one classification round, where the image sequence is extended in 10-day increments from May to October. The green bars indicate the imagery coverage period used in each round, while the blue extensions show the additional time span of images progressively included. The red arrows connect each temporal sequence to its corresponding classification accuracy result. The bottom axis shows the soybean growth calendar (sowing, seeding, trifoliation, flowering, pod setting, and maturity/harvest).

To examine how soybean recognition accuracy varies throughout different stages of crop development, this study simulates classification performance over time by defining a series of image sequences with progressively extended temporal coverage. As illustrated in **Figure 8**, the start date is fixed at May 1st, representing the early sowing period, while the end date is incrementally extended in 10-day intervals until October 8th, corresponding to crop maturity and harvest. This results in a total of 14 experimental rounds. In each round, Sentinel-1 and Sentinel-2 imagery within the specified timeframe is used to construct a time series, which is then analyzed using the proposed classification method to identify soybean cultivation and assess recognition accuracy. Due to limited data availability in early stages, the first experimental round covers the period from May 1st to May 31st. As the season progresses, the image sequence becomes increasingly complete, leading to gradual improvements in recognition accuracy. At a certain point, accuracy reaches a practical threshold that allows for early-season mapping. To determine this threshold—referred to as the earliest identifiable time (EIT)—this study adopts the criterion proposed by You and Dong (2020), whereby the F1 score for soybean classification first exceeds 0.9. Moreover, this experiment compares the temporal evolution of classification accuracy across three data input strategies—optical only, SAR only, and multi-source fusion—highlighting the advantages of multi-source integration at various phenological stages. This dynamic assessment of accuracy under both crop growth and time-series progression underscores the value of multi-source data in improving both the precision and timeliness of soybean identification.

**Figure 9:**
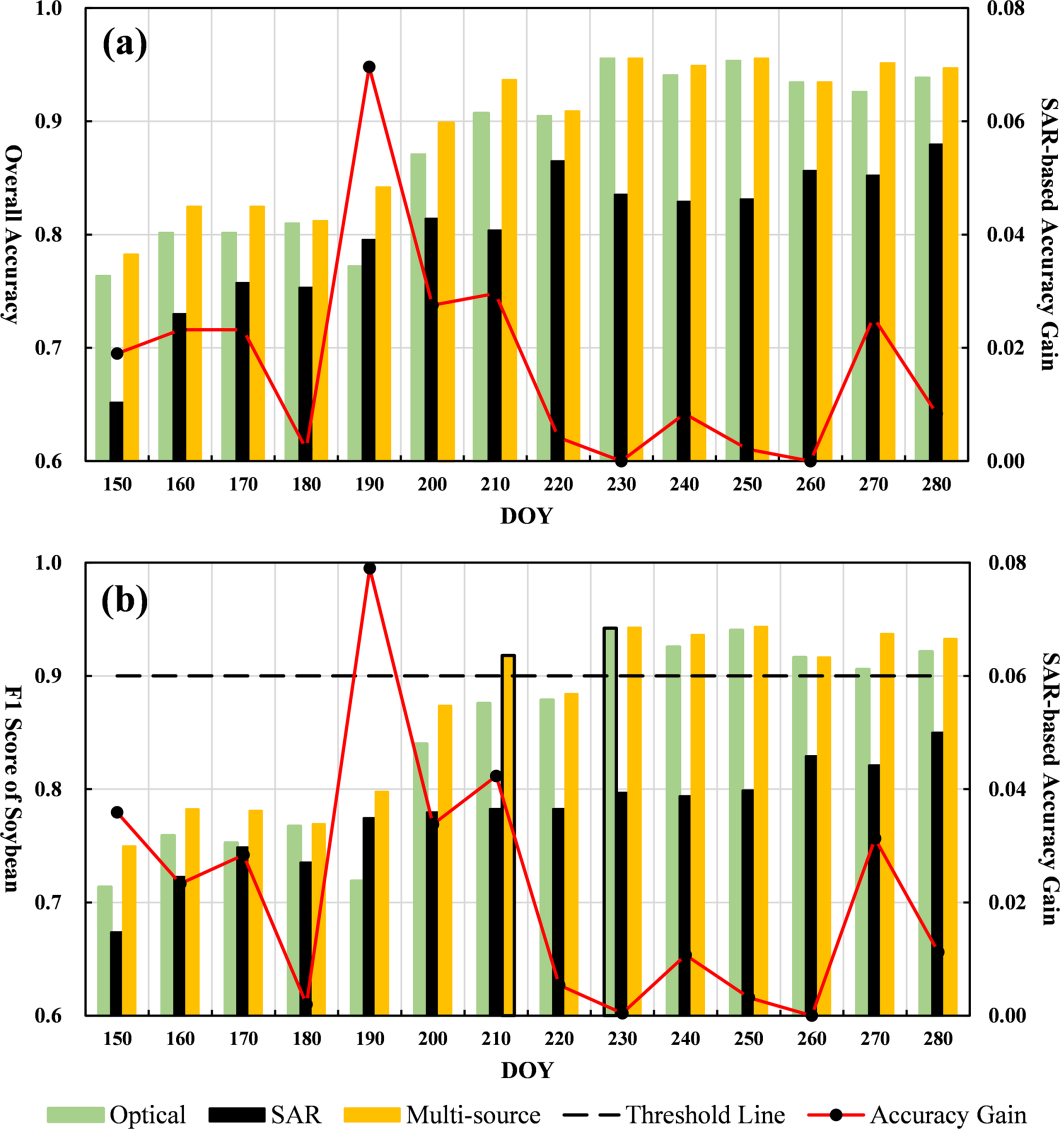
Temporal progression results: **(a)** Bar chart of OA for three data sources with red line indicating SAR contribution; **(b)** Bar chart of soybean F1 scores with red line for SAR contribution and black dashed line representing the F1 = 0.9 threshold.

As shown in **Figure 9**, soybean recognition accuracy is closely linked to the crop’s phenological development. As growth progresses, both overall accuracy (OA) and the F1 score steadily increase. When segmented by phenological stages, average classification performance improves accordingly: during the sowing period (DOY ≤ 150), OA reaches 78.27% and F1 score is 74.94%; during the three-leaf stage (DOY 160–180), OA rises to 82.07%, and F1 to 78.22%; in the flowering period (DOY 190–210), OA improves to 89.23%, and F1 to 86.32%; during the pod-setting stage (DOY 220–240), OA climbs to 93.80%, and F1 reaches 92.09%; and by the grain-filling and maturity stage (DOY 250–280), OA reaches 94.72%, with an F1 score of 93.23%. Accuracy gains become less pronounced in the later stages.

The most substantial improvements in accuracy occur during the flowering and pod-setting stages. These phases correspond to vigorous vegetative growth, canopy closure, and enhanced physiological differentiation, making crop-specific spectral and structural features more detectable. This pattern aligns with the feature importance analysis, which indicates a higher concentration of influential features during these periods. In early stages—such as sowing and the three-leaf phase—soybean plants remain short with sparse canopy cover, and imagery primarily captures bare soil, resulting in lower recognition accuracy. By the three-leaf stage, young stems emerge but the canopy remains underdeveloped, producing modest accuracy gains (OA and F1 increase by approximately 3.80% and 3.28%, respectively). The flowering period marks a critical point for yield formation, as branching intensifies and canopy cover becomes complete. Both optical and SAR features are more pronounced, leading to sharper accuracy gains: OA and F1 improve by 7.16% and 8.10% over the previous stage. During pod-setting, continued physiological development and pod enlargement further enhance separability, yielding an additional OA increase of 4.57% and F1 increase of 5.77%. In the grain-filling and maturity stages, soybean senescence leads to spectral stabilization, resulting in only slight accuracy improvements—0.92% in OA and 1.14% in F1.

When comparing classification performance across different data sources throughout crop growth, multi-source fusion consistently achieves the highest accuracy, demonstrating superior robustness and adaptability. Accuracy improves gradually in the early season and increases sharply after flowering, plateauing around DOY 230 (mid-pod-setting). Optical data performs similarly to multi-source data across most stages and remains the primary source for remote sensing classification. However, SAR-only data, despite generally lower accuracy, performs particularly well during critical stages—especially under conditions of poor optical image quality due to frequent cloud cover. Notably, around DOY 190, SAR-based accuracy temporarily surpasses that of optical data, highlighting its potential for independent classification in cloudy conditions. **Figure 9a** further quantifies SAR’s complementary role. During the sowing and three-leaf stages, SAR contributes modest OA improvements (~2–3%) due to limited optical information. As soybeans enter flowering, SAR’s added value increases significantly, peaking at a 6.69% OA improvement on DOY 190. This contribution declines during pod-setting and grain-filling stages, as optical features become more distinctive and the marginal benefit of SAR diminishes. Analysis of the F1 score trends in **Figure 9b** reveals consistent patterns across data sources: slow improvement in early stages, rapid gains from flowering to pod-setting, and eventual stabilization. Multi-source fusion consistently yields the highest F1 score, followed by optical, with SAR alone performing lowest. Around DOY 200 (mid-flowering stage), F1 scores for optical and multi-source data surge as soybean canopy and pod characteristics become prominent. Compared to the previous round, the F1 score for optical data increases by 12.88%, and for multi-source data by 7.59%. SAR’s F1 score reaches 77.45% on DOY 190 and gradually peaks at 84.99% by DOY 280. These trends reinforce the supplementary value of SAR. From sowing to the three-leaf stage, SAR contributes approximately 3% to the F1 score. During the flowering stage, this contribution increases to a peak of 6.96%, before declining in the later stages as optical information becomes dominant.

The EIT for soybean using multi-source data is DOY 210 (early pod-setting stage), when canopy structures become clearly distinguishable and spectral features are well defined. At this point, the F1 scores for multi-source, optical, and SAR data are 91.80%, 87.57%, and 78.24%, respectively. Compared to the optical-only strategy, multi-source fusion advances the EIT by approximately 20 days, with a 4.23% improvement in F1 score. In contrast, optical data reaches an F1 score of 90% at DOY 230 (late pod-setting to early maturity), while SAR data never achieves the EIT threshold throughout the season.

In summary, incorporating SAR data significantly advances the EIT by approximately 20 days, enhancing the timeliness of soybean mapping. This earlier detection capability provides valuable lead time for downstream applications such as yield forecasting, pest and disease monitoring, seasonal planning, and agricultural decision-making.

### 3.4 Socio-economic implications of early and accurate crop identification

Early and accurate crop identification provides significant economic and social benefits across multiple scales. At the national and regional levels, timely information on crop distribution and growth conditions enhances yield forecasting and monitoring, offering governments and markets more reliable production and supply expectations to support food security decisions and emergency responses such as reserve management and relief distribution (Hoefsloot et al., 2012; Setiyono et al., 2014). At the farm level, detailed crop maps enable more efficient allocation of inputs such as fertilizers, seeds, and irrigation, while guiding key management decisions on sowing, irrigation, and pest control, thereby improving resource-use efficiency, reducing yield losses, and stabilizing farmers’ incomes (Wu et al., 2023). Beyond farm management, remote sensing–derived indicators of crop cover and dynamics are increasingly applied in agricultural insurance, where they function as claim triggers and inputs for premium pricing, improving both the efficiency and sustainability of insurance systems. When combined with crop modeling, these data further strengthen the transparency and credibility of multi-risk assessments (Basso et al., 2013; Liu et al., 2025). From a broader macroeconomic perspective, reliable early assessments help reduce market uncertainty, stabilize supply chains, and inform price forecasting. Such high-quality information has been shown to mitigate global market shocks (Tanaka et al., 2023) and strengthens national food security strategies by providing spatially and temporally refined data for timely and targeted policy responses (Zhang et al., 2025).

## 4 Conclusion

This study addresses the persistent challenges of spectral confusion and data incompleteness in soybean remote sensing classification by proposing a multi-source approach that integrates Sentinel-1 SAR and Sentinel-2 optical time-series imagery. Through systematic evaluation, we demonstrate that multi-source data fusion significantly enhances both the accuracy and timeliness of soybean identification. The integration of optical and SAR data yielded the highest classification performance, achieving an overall accuracy (OA) of 96.85%, a Kappa coefficient of 0.9493, a soybean F1-score of 95.84%, and an area accuracy of 96.57%. While the improvement over optical data alone was relatively modest—raising the F1-score and area accuracy by 0.81% and 0.83%, respectively—the improvement over SAR-only classification was substantial. This highlights the strong baseline performance of optical data and the complementary role of SAR, particularly under cloud-contaminated or spectrally ambiguous conditions, where SAR data significantly contributes to classification robustness. Soybean classification accuracy improved progressively with crop development. During the sowing stage, weak spectral signals led to low recognition accuracy. As soybeans entered the flowering and pod-setting stages, canopy closure and physiological changes enhanced both spectral and structural separability, resulting in rapid gains in classification accuracy. The F1-score increased from 78.22% in July to 92.09% in August. Notably, SAR data provided the greatest incremental value during the flowering and pod-setting stages, with accuracy gains of up to 6.96%. In contrast, during the grain-filling and maturity stages, the contribution of SAR diminished as optical signals became more distinctive and stable. Furthermore, multi-source data advanced the EIT for soybean to DOY 210, approximately 20 days earlier than when using optical data alone. This temporal advancement is crucial for early-season applications such as yield forecasting, pest and disease monitoring, and harvest scheduling.

In summary, the integration of multi-source time-series remote sensing and the targeted extraction of phenological and key-stage features constitute an effective strategy for high-precision and early soybean identification. The methodology proposed in this study provides both theoretical insights and practical tools for large-scale agricultural monitoring.

## Data Availability

Publicly available datasets were analyzed in this study. This data can be found here: The Sentinel-1 and Sentinel-2 imagery used in this study is publicly available from the Copernicus Open Access Hub: https://scihub.copernicus.eu/ Preprocessing and time-series analysis were performed via Google Earth Engine (https://earthengine.google.com/) using freely accessible Sentinel datasets. Repository: Copernicus Open Access Hub Accession number(s): Not applicable (data are accessed by geographic coordinates and date range).
